# *De novo* transcriptome assembly and annotation of the common freshwater amphipod (*Gammarus pulex*) a valuable resource for ecotoxicogenomics

**DOI:** 10.1038/s41597-025-05872-2

**Published:** 2025-08-28

**Authors:** Camilo Escobar-Sierra, Sameer Hassan, Fabian G. Weichert, Henrik Aronsson, Kathrin P. Lampert, Henner Hollert, Thomas Backhaus, Pedro A. Inostroza

**Affiliations:** 1 Institute for Environmental Research, RWTH Aachen University, Aachen, Germany; 2 Institute of Zoology, University of Cologne, Cologne, Germany; 3 Department of Biological and Environmental Science, University of Gothenburg, Gothenburg, Sweden; 4 Department of Evolutionary Ecology & Environmental Toxicology (E3T), Faculty of Biological Sciences, Goethe University Frankfurt, Frankfurt, Germany; 5 Department of Environmental Media-Related Ecotoxicology, Fraunhofer Institute for Molecular Biology and Applied Ecology (IME), Frankfurt am Main, Germany; 6 LOEWE Centre for Translational Biodiversity Genomics (LOEWE-TBG), Frankfurt am Main, Germany

**Keywords:** Transcriptomics, Pollution remediation, Environmental monitoring

## Abstract

This study presents *de novo* transcriptome assemblies for *Gammarus pulex*, a freshwater amphipod widely used in ecotoxicology due to its ecological importance and sensitivity to pollution. Specimens were collected from 13 river sites in Germany and Sweden, encompassing a gradient of micropollutant exposure. Using high-throughput RNA sequencing, we generated transcriptomes for German, Swedish, and combined populations. The assemblies yielded up to 170,000 transcripts with strong metrics, including N50 values over 1,500 base pairs and completeness scores approaching 89%. Functional annotation revealed over 123,000 unique protein hits, nearly 99,000 BLASTx matches, and approximately 30,000 annotated KEGG pathways. We also identified thousands of conserved domains, signal peptides, and transmembrane proteins. These comprehensive resources provide valuable molecular insight into the stress responses of *Gammarus pulex* and will facilitate the development of gene-based biomarkers for freshwater monitoring. By improving the molecular toolkit for this key sentinel species, our study supports broader applications of ecotoxicogenomics in environmental assessment and conservation.

## Background & Summary

Ecotoxicogenomics is an emerging field that integrates genomics-based tools (such as genomics, transcriptomics, and proteomics) to investigate the molecular effects of environmental stressors, with applications ranging from model species to non-target and non-model organisms^1^. It provides valuable insights into the molecular mechanisms underlying responses to pollutants, offering potential biomarkers for monitoring the ecological status of aquatic environments. Invertebrates, particularly crustaceans, play a pivotal role in aquatic ecosystems due to their positions in food webs and their sensitivity to natural and chemical stressors^2^. Gammarids have long been used as model organisms in ecotoxicology due to their ecological importance, wide distribution, and sensitivity to pollutants. The common freshwater amphipod, *Gammarus pulex*, has gained considerable attention in ecotoxicology studies as a non-target and non-model organism due to its wide distribution^3^, ecological relevance^4,5^, and susceptibility to emerging chemicals^2,6,7^. Despite this, genomic resources as transcriptomes or genomes for *Gammarus pulex* (and gammarids in general), remain limited, hindering deeper insights into their molecular responses to environmental stress. Existing transcriptome assemblies^8,9^ are based on restricted sample sizes or specific tissues, and report lower completeness and annotation quality. In contrast, the present study provides a multi-population transcriptome resource for *Gammarus pulex* that addresses these limitations. Although a fragmented genome exists for *Gammarus lacustris*^10^ and a reference genome is available for *Hyalella azteca*^11^, no high-quality genome is currently available for *Gammarus pulex*, emphasizing the need for comprehensive molecular resources for this ecologically important taxon.

The development of transcriptomic resources for non-model species like *Gammarus pulex* is essential to enhance our ability for environmental monitoring. Transcriptome data, which provides a snapshot of gene expression, is particularly valuable for understanding organismal responses to environmental stressors and chemical pollution^12^. Unlike genomic data, transcriptomics focuses on the expressed portion of the genome, allowing the identification of genes that are actively involved in stress responses^13^. Moreover, generating high-quality transcriptome resources for species lacking published reference genomes not only facilitates the identification and interpretation of differentially expressed genes (DEGs) but also enables the use of streamlined workflows that do not rely on replicating a computationally demanding *de novo* assembly. Transcriptome assembly and annotation provide a foundation for gene expression analysis, enabling the identification of DEGs linked to environmental stress. These DEGs can act as molecular biomarkers of exposure and effects, even revealing the underlying molecular mechanisms of ecotoxicity. Furthermore, transcriptomic databases provide complementary information that is especially useful for genome annotation and insights into gene functioning^14^. As more transcriptome profiles are archived, they will allow for enhanced meta-analytical approaches, improving our ability to elucidate specific functional response pathways and providing crucial insights into baseline gene expression profiles and stress responses.

A key challenge in ecotoxicology is linking molecular biomarkers to higher-level ecological effects. The Adverse Outcome Pathway (AOP) framework provides a structured approach to bridging this gap by connecting molecular initiating events (MIEs) to adverse outcomes pathways (AOPs) through key events (KEs) and key event relationships (KERs)^15^. By integrating transcriptomic data within this framework, researchers can establish mechanistic links between molecular disruptions and organismal or population-level effects, offering a predictive and regulatory-relevant perspective on ecotoxicological impacts^16^. The integration of AOPs with transcriptomic data is particularly valuable in non-model organisms like *Gammarus pulex*, whose ecological significance and sensitivity make it an ideal species for investigating molecular responses to stressors. For instance, transcriptomic insights can help identify MIEs related to endocrine disruption, immune suppression, or metabolic dysfunction, which, through a cascade of key events, could lead to population declines or ecosystem instability. Furthermore, identifying stress-responsive genes involved in detoxification pathways or oxidative stress responses can support biomonitoring efforts by providing early-warning indicators of pollution exposure. Additionally, understanding the genetic basis of pollutant tolerance may help identify populations with higher adaptive potential, informing conservation and restoration strategies.

*Gammarus pulex* plays a crucial ecological role as a detritivore, contributing to the breakdown of organic matter in freshwater ecosystems^4,5^. Its sensitivity to pollutants, including metals^17^, pesticides^18^, pharmaceuticals^19^, and complex mixtures of micropollutants^2,6,20^, makes it an excellent sentinel species for assessing the health of aquatic ecosystems. Wastewater treatment plants (WWTPs) have been shown to negatively impact *Gammarus pulex* populations, causing decreased feeding rates^20^, increased mutation rates^6^, and oxidative stress^21^. These studies have primarily focused on behavioural and physiological endpoints (e.g., feeding rates, reproduction, and survival). However, such phenotypic observations offer limited insight into the underlying molecular mechanisms of stress responses. So far, molecular and physiological analyses in *Gammarus pulex* have been hampered by a lack of omics data^8,22^. Although widely used in environmental monitoring, *Gammarus pulex* still lacks comprehensive molecular resources such as high-quality transcriptomes, limiting mechanistic ecotoxicological studies compared to established model invertebrates like *Drosophila melanogaster* or *Caenorhabditis elegans*.

To address the current gap and enhance the application of transcriptomics in ecotoxicogenomics, this study focuses on generating three *de novo* transcriptome assemblies for *Gammarus pulex* using RNA sequencing (RNA-Seq). *Gammarus pulex* were collected in streams and rivers characterised by complex mixtures of micropollutants including pesticides, pharmaceuticals, and industrial chemicals and areas with low micropollution pressure in Sweden^23,24^ and Germany^25–27^. The objectives are threefold: (1) to assemble high-quality transcriptomes for each of the sampling areas, and a joint assembly using the entire dataset, (2) to annotate the assembled transcripts with functional information, and (3) to assess the completeness and quality of the transcriptome assemblies and annotation. These resources are expected to not only advance molecular research on *Gammarus pulex* but also provide actionable data for integrating transcriptomic insights into biomonitoring programs and AOP-based regulatory frameworks. By providing comprehensive transcriptome resources for *Gammarus pulex*, this work will support future ecotoxicogenomics research, enabling the identification of molecular markers for environmental monitoring and a better understanding of the species’ molecular response to environmental stressors.

## Methods

### Ethic statement

No specific permits were required to collect *Gammarus pulex* from the different sampling sites. They are not on any list of endangered or protected species.

### Study area and sample collection

**Fig. 1:**
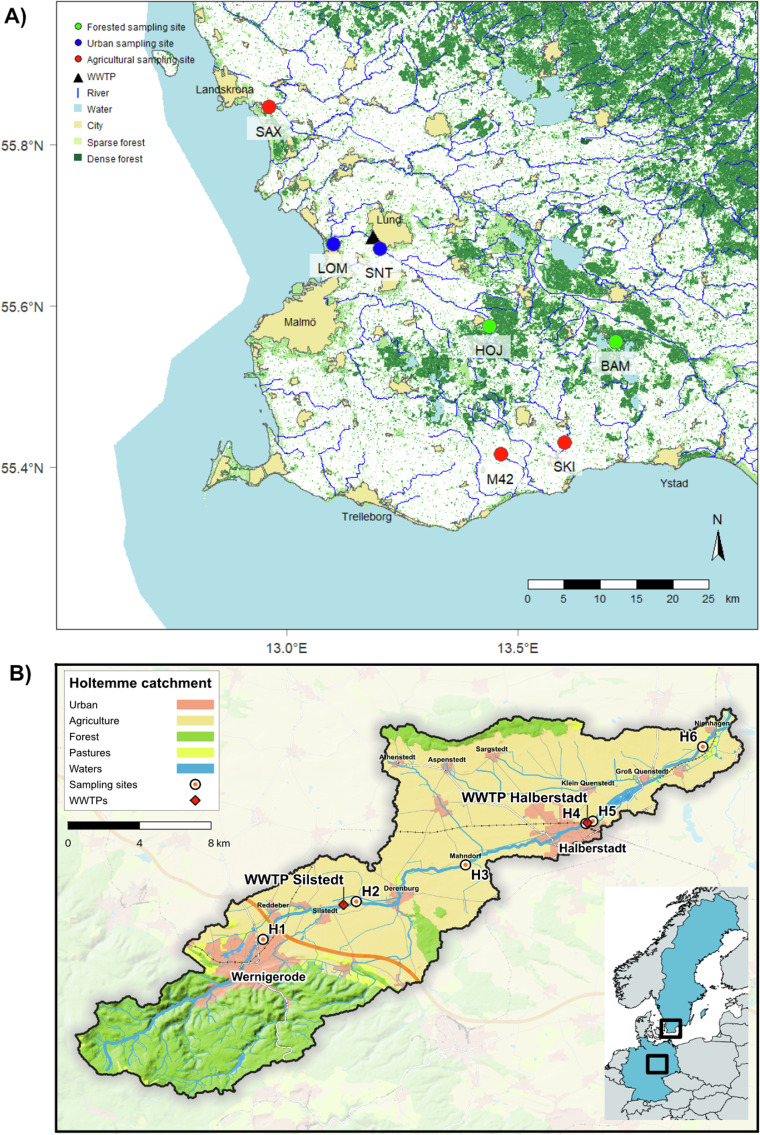
Maps showing the location of the sampling sites. (**A**) Sweden and (**B**) Germany (B). Land uses are also summarised in the legend.

Thirteen sampling sites, located in streams and small rivers across Southern Sweden and Central Germany, were selected for *Gammarus pulex* collection (Fig. 1). These sites represented distinct land-use patterns and micropollutant profiles, facilitating a comprehensive analysis of gene expression responses. In Southern Sweden, characterised by intensive agriculture and the Malmö-Lund metropolitan area, two sites (HOJ and BAM) within natural reserves served as low-anthropogenic-pressure references. Three sites (M42, SKI, and SAX) were situated in agricultural regions, while two (SNT and LOM), located upstream and downstream of Lund’s wastewater treatment plant (WWTP), reflected urban influences. Similarly, in the River Holtemme (Central Germany), one site (H1), upstream of Silstedt’s WWTP, represented low anthropogenic pressure, two (H3 and H6) were in agricultural areas, and three (H2, H4, and H5), including two downstream of WWTPs, captured urban impacts. Previous micropollutant characterization and risk assessment studies in both regions have highlighted potential risks to aquatic invertebrates^28,29^.

*Gammarus pulex* specimens were sampled following a standardised sampling protocol^30^ in the summer of 2021 (Germany) and 2022 (Sweden). Twenty habitat-weighted samples were taken from a total area of 1 m^2^ at each site with a Surber sampler (500 μm mesh size). After collection, specimens were flash-frozen on dry ice or a cryoshipper (Voyageur-12, Air Liquide) and stored at −80 °C until RNA isolation, eliminating the need for RNAlater. In total, RNA was extracted from 65 specimens (5x specimen per sampling site), from 5 rivers/small streams in Skåne region in Sweden and the River Holtemme (Saxony-Anhalt) in Germany. Stream and/or river names and respective geocoordinates are listed in Supplementary Table 1.

### RNA extraction, library preparation and sequencing

Samples were crushed in liquid nitrogen using a mortar and pestle, and total RNA was extracted individually from each specimen using the RNeasy Plus Mini Kit (Qiagen, Germany) according to the manufacturers’ instructions. In addition, genomic DNA was removed using DNAse I (ThermoFisher Scientific, Germany). RNA was eluted in 60 µL RNAse-free water, which was passed twice through the spin column to maximise the concentration of eluted RNA. The quantity and quality of extracted RNA were assessed using the Qubit RNA Assay Kit in a Qubit 2.0 Flurometer (ThermoFisher Scientific) and NanoDrop 2000c (ThermoFisher Scientific), respectively. RNA Integrity Number (RIN) was measured using a 2200 TapeStation (Agilent Technologies, Germany) and only samples with RIN > 7 were sequenced.

Library preparation and sequencing were performed by Novogene (Cambridge, United Kingdom). Briefly, messenger RNA (mRNA) was purified from 3 µg total RNA using poly-T oligo-attached magnetic beads (Invitrogen, USA). The RNA strands were subsequently fragmented with divalent cations in NEB First Strand Synthesis reaction buffer (NEB, USA), followed by first- and second-strand cDNA synthesis. The library was ready after end repair, A-tailing, adapter ligation, size selection, amplification, and purification. The library was checked with Qubit 2.0, real-time PCR for quantification, and 2100 BioAnalyzer (Agilent) for size distribution detection. Quantified libraries were pooled and sequenced on Illumina NovaSeq. 6000, generating 150 bp paired-end reads.

### *De novo* transcriptome assembly, refinement, and quality assessment

**Fig. 2:**
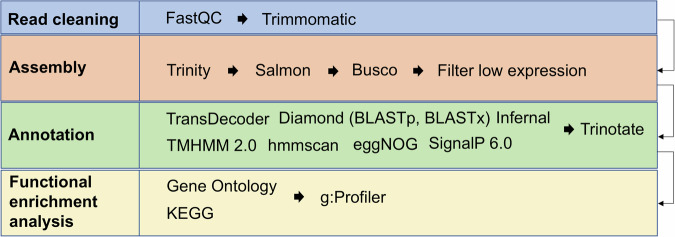
Workflow chart of the bioinformatic pipeline. Starting from the read cleaning of the raw data to the annotation of the *de novo* transcriptomes.

The bioinformatic workflow for transcriptome assembly and annotation is illustrated in Fig. 2. A total of 30 paired-end libraries were processed for the German transcriptome (DE), 35 for the Swedish transcriptome (SWE), and 65 for the combined DE-SWE transcriptome. Initial quality control of the raw reads was performed using FastQC^31^ to evaluate sequence quality. Following this, low-quality reads and adapter sequences were removed with Trimmomatic^32^, using a quality threshold of phred + 33 and ensuring a minimum base quality score of 25. Reads shorter than 50 bp were also filtered out to improve downstream analysis accuracy.

The Trinity v2.9.1^33^ assembler was employed to generate each dataset’s *de novo* transcriptome assemblies. Trimmed reads were subsequently mapped back to the assembled transcriptome using Salmon^34^, which was used to estimate transcript abundance. We first used the script TrinityStats.pl contained in TRINITY for assembly quality assessment, followed by BUSCO v5.7.2^35^, which evaluated assembly completeness by comparing the results against the Arthropoda Odb10 datasets (updated January 2024). An expression matrix was generated, and low-expressed transcripts (TPM < 1.0) were removed using Trinity’s native functions. The expression levels were then normalized to transcripts per million (TPM) to standardize transcript quantification across samples.

### Transcriptome annotation

Annotation was performed to identify protein-coding regions using TransDecoder v5.7.0^33^ to predict open reading frames (ORFs). Functional annotation of the transcriptomes involved aligning the filtered transcripts to protein databases using DIAMOND v2.0.8^36^ for sequence alignment and identifying signal peptides and transmembrane domains with SignalP 6.0^37^ and TMHMM v2.0^38^, respectively. Additionally, HMMER v3.3.2^39^ was used to search for conserved protein domains, and Infernal 1.1.5^40^ identified non-coding RNA features. Additional gene annotation was performed using eggNOG-mapper v2.1.1^41^ with the eggNOG 5.0^42^ database. The results from these tools were integrated into a local SQLite database using the Trinotate v4.0.2^43^ pipeline, which produced a comprehensive functional annotation of the transcriptomes. Finally, gene lists from the BLASTp and BLASTx outputs were used to perform an overrepresentation analysis of functional pathways using the Gene Ontology (GO) and KEGG databases, facilitated by the g:Profiler web application^44^.

### Statistics

Trinotate outputs were statistically summarised using TrinotateR package (https://github.com/cstubben/trinotateR) implemented in R version 4.3.0.

## Data Records

The raw RNA-seq reads from *Gammarus pulex* individuals collected at 13 sampling sites in Germany and Sweden are publicly available in the NCBI Sequence Read Archive (SRA) under accession number SRP571512, accessible via the persistent identifier: http://identifiers.org/dbest:SRP571512^45^. Transcriptome assemblies were deposited in the NCBI Transcriptome Shotgun Assembly (TSA) Sequence Database under accession numbers GLGZ00000000^46^ (version GLGZ01000000), GLHA00000000^47^ (version GLHA01000000), and GLHB00000000^48^ (version GLHB01000000), corresponding to the German (DE), Swedish (SWE), and combined (DE_SWE) transcriptomes, respectively.

All additional data resources—including *de novo* transcriptome assemblies (FASTA), predicted amino acid sequences (PEP), normalized expression matrices (TABULAR), functional annotations (TSV and structured text), transcriptome completeness metrics from BUSCO (TXT, GFF3, and TABULAR), and high-resolution figures (TIFF)—are organized by assembly (DE, SWE, DE_SWE). Each assembly has subdirectories containing folders for annotation, assembly, BUSCO validation, and expression data in our Figshare repository 10.6084/m9.figshare.28451624.v3^49^. Supplementary Table 1, which lists each RNA-seq library with its internal sample name, collection date, geographic coordinates, and corresponding SRA run accession (SRR), BioSample accession (SAMN), and Experiment accession (SRX), provides essential metadata for reproducibility and reuse.  

## Technical Validation

Validation of transcriptome completeness and annotation quality. To assess the quality and functional breadth of the assembled transcriptomes, we incorporated Gene Ontology (GO) and KEGG pathway enrichment analyses as part of the annotation validation process. These analyses were not used to interpret biological outcomes or test specific hypotheses, but to verify that key cellular functions and core metabolic pathways were well represented, indicating successful transcriptome assembly and annotation. Differences in functional categories among DE, SWE, and DE-SWE assemblies further reflect annotation depth and transcript diversity, supporting their value as reusable data resources.

### Quality of the raw reads and transcriptome validation

Each of the three assemblies (DE, SWE, DE-SWE) was filtered to ensure high data quality, with summary statistics provided in Table 1. Filtering reduced the total assembled bases substantially in each assembly: DE decreased by 75.8%, SWE by 69.7%, and DE-SWE by 69.1%, indicating the effectiveness of the quality measures. Consequently, the total number of Trinity genes and transcripts also decreased, with DE genes reduced by 64.8%, SWE by 55.5%, and DE-SWE by 57.3%, producing a curated set of contigs. The DE-SWE combined assembly retained the largest number of genes and transcripts post-filtering, indicative of higher sequence diversity in the combined dataset.

**Table 1:** Statistics of the *de novo* assemblies before and after quality filtering.

	DE		SWE		DE-SWE	
	Raw	Filtered	Raw	Filtered	Raw	Filtered
Total assembled bases:	1,136,699,311	275,494,270	1,283,708,987	389,068,549	1,654,435,040	511,664,251
Total trinity ‘genes’:	1,267,578	446,047	1,331,455	591,607	2,113,736	903,272
Total trinity transcripts:	1,693,841	545,059	1,893,121	758,632	2,811,218	1,123,011
Percent GC:	42.10	42.52	43.12	43.28	42.17	42.70
Contig N10:	4,786.00	4,425.00	4,476.00	3,862.00	3,413.00	3,164.00
Contig N50:	997.00	703.00	1,488.00	707.00	752.00	476.00
Average contig:	671.08	505.44	678.09	512.86	588.51	455.62

The DE column represents the results for the German assembly, SWE for the Swedish assembly, and DE-SWE for the combined assembly.

**Fig. 3:**
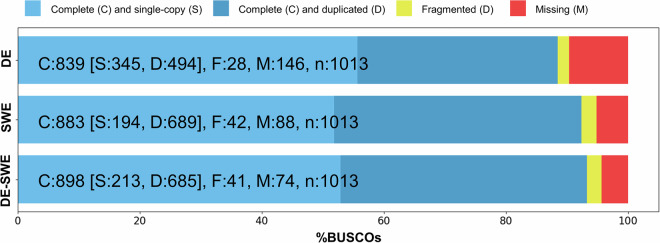
BUSCO completeness assessment of the three transcriptomes using the Arthropoda Odb10 dataset. Colours represent different BUSCO categories, with percentages indicating the proportion of each category within the total BUSCO groups analysed.

GC content remained relatively consistent, with minor increases: DE increased from 42.1% to 42.5%, SWE from 43.1% to 43.3%, and DE-SWE from 42.2% to 42.7%, suggesting that quality control did not significantly alter the base composition. Contig N10 and N50 values were also reduced, reflecting the retention of shorter, high-confidence contigs. Average contig lengths also decreased across each transcriptome after filtering, with DE showing a reduction of 24.7%, SWE by 24.4%, and DE-SWE by 22.6%, aligning with the expected outcome of removing low-confidence, longer contigs. Following this quality filtering, a BUSCO completeness assessment of each transcriptome confirmed high completeness levels across all filtered assemblies (Fig. 3), underlining the robustness of these datasets for downstream analyses.

### Quality of the transcriptome annotation

Annotation was achieved for all three transcriptome assemblies (DE, SWE, and DE-SWE), with results summarized in Table 2. Each transcriptome was annotated using various databases, capturing various functional and structural features. The DE assembly produced 545,059 unique transcripts, SWE yielded 758,632, and the combined DE-SWE assembly comprises 1,123,011 unique transcripts. BLASTx hits revealed 48,506 unique annotations for DE (8.9% of transcripts), 57,928 for SWE (7.6%), and 98,785 for DE-SWE (8.8%), with GO annotation following similar trends, suggesting that combined data increases annotation depth. Protein hits also scaled with assembly size, with DE yielding 59,958 unique protein hits, SWE 75,331, and DE-SWE 123,828. Transmembrane regions and signal peptides were more prevalent in larger assemblies, with DE-SWE displaying the most diversity (6,815 transmembrane regions and 16,472 signal peptides). Functional pathway annotation via KEGG was highest in DE-SWE (29,894 unique hits), reflecting enriched metabolic diversity, with similar increases seen in BLASTp and Pfam annotations across assemblies. EggNOG and non-coding RNA annotations followed the same pattern, with DE-SWE capturing the broadest range. Overall, the combined DE-SWE assembly displayed the highest functional and structural diversity.

**Table 2:** Summary of gene annotation hits from different tools.

Feature	DE		SWE		DE-SWE	
	Unique	Total	Unique	Total	Unique	Total
Transcripts	545,059	545,059	758,632	758,632	1,123,011	1,123,011
BLASTx hits	48,506	49,407	57,928	59,003	98,785	100,558
BLASTx GO	17,980	49,074	19,232	58,523	27,010	99,838
Proteins	59,958	66,296	75,331	83,892	123,828	133,595
Transmembrane regions	4,215	66,296	4,472	83,892	6,815	133,595
KEGG	18,902	38,741	20,537	46,581	29,894	79,525
BLASTp hits	23,443	27,040	27,493	32,168	47,302	52,517
BLASTp GO	10,826	26,870	11,376	31,934	17,194	52,171
Pfam hits	29,623	34,104	33,659	39,680	57,639	64,454
Signal peptides	10,294	66,296	12,514	83,892	16,472	133,595
eggnog	790	1,725	1,073	2,464	1,246	3,770
non-coding RNA	1,181	1,194	1,597	1,628	2,586	2,623

The DE column presents results for the German assembly, SWE for the Swedish assembly, and DE-SWE for the combined assembly.

**Fig. 4:**
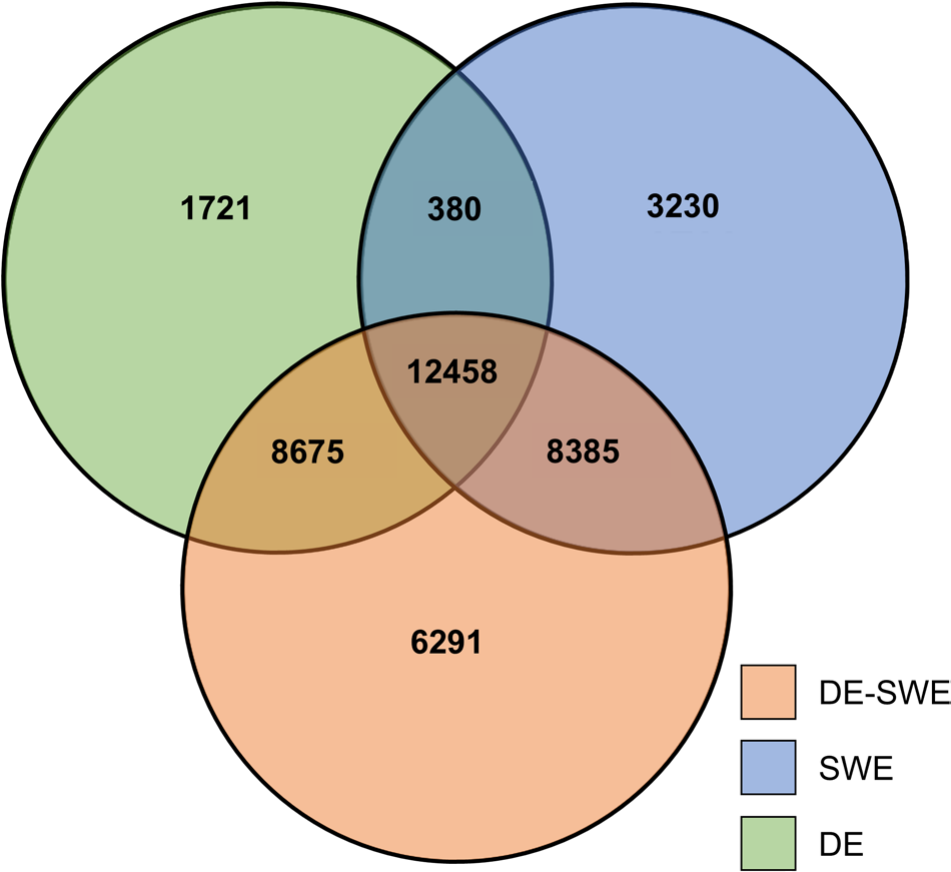
Overlap of annotated genes across assemblies as a measure of coverage and diversity. A Venn diagram illustrates the core set of shared annotated genes and unique subsets in each assembly, supporting the increased depth provided by the combined DE-SWE transcriptome.

The annotation of the three *de novo* transcriptomes revealed a core set of 12,458 shared annotated genes, while each assembly retained unique subsets: 1,721 genes were exclusive to the Germany (DE) transcriptome, 3,230 to the Sweden (SWE) transcriptome, and 6,291 to the combined DE-SWE transcriptome. A Venn diagram (Fig. 4) illustrates these overlaps, highlighting both conserved and unique annotations. The DE-SWE assembly demonstrated the highest completeness, sharing 8,675 genes with DE and 8,385 with SWE, likely due to increased sequencing depth, improved transcript reconstruction, and broader representation of population-specific expression. Its higher number of unique genes reflects enhanced detection of lowly expressed or rare transcripts, better isoform resolution, and reduced assembly fragmentation. These outcomes further confirm the suitability of the dataset as a reference transcriptome for functional studies.

### Taxonomic validation of annotation

**Fig. 5:**
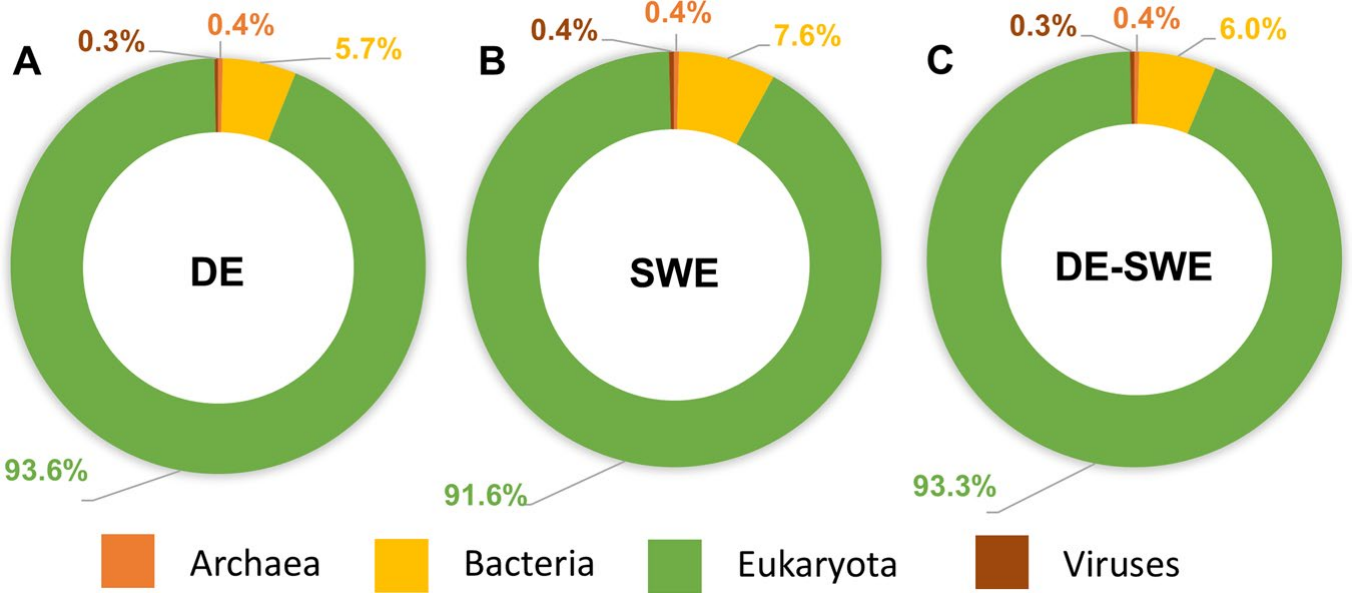
Top-level taxonomic assignments from SwissProt BLASTp hits as a quality control check. Panels (**A–C**) show results for the German (DE), Swedish (SWE), and combined (DE-SWE) transcriptomes, respectively.

**Fig. 6:**
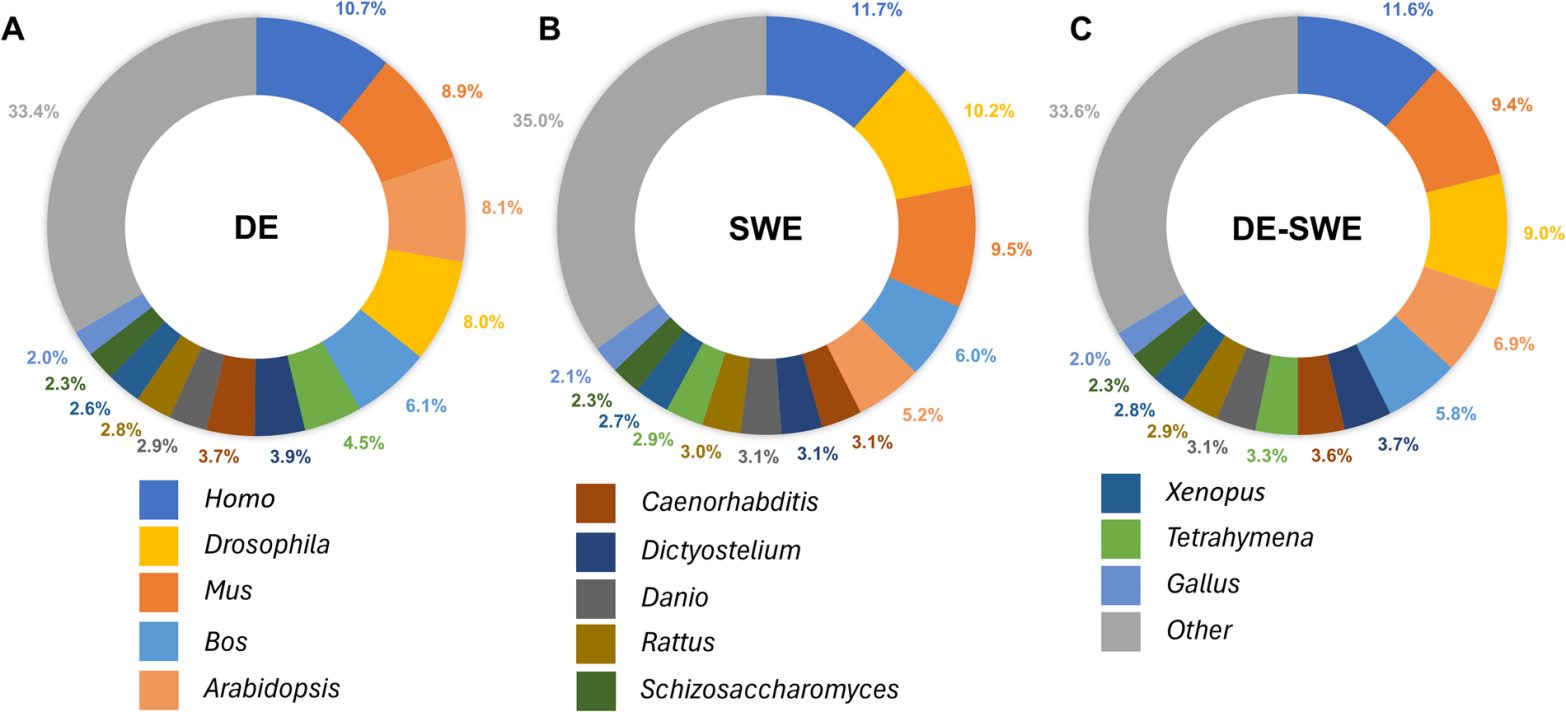
Most abundant genera in SwissProt annotations for transcriptome validation. Top genera and total genus-level diversity are shown for DE, SWE, and DE-SWE assemblies.

The analysis of taxonomic assignments in the three transcriptome assemblies revealed a consistent pattern across all samples when compared against the SwissProt database (Fig. 5). Over 90% of the hits for each assembly were attributed to Eukaryota, with the DE assembly showing 37,354 hits (93.6%), SWE 41,352 hits (91.6%), and the DE-SWE combined assembly exhibiting 77,701 hits (93.3%). Bacterial hits accounted for 5 to 7% of the total, with DE having 2,286 hits (5.7%), SWE 3,419 hits (7.6%), and DE-SWE 5,006 hits (6.0%). In contrast, Archaea and Viruses represented only marginal percentages, comprising less than 1% of the hits across all assemblies. When assessing the genus level, a similar pattern was found for all three assemblies, with top hits belonging to the genus *Homo*, *Bos*, *Mus*, *Drosophila* and *Arabidopsis* (Fig. 6). These findings provide additional quality control evidence and confirm the expected taxonomic composition of the dataset.

### GO enrichment to assess annotation breadth

**Fig. 7:**
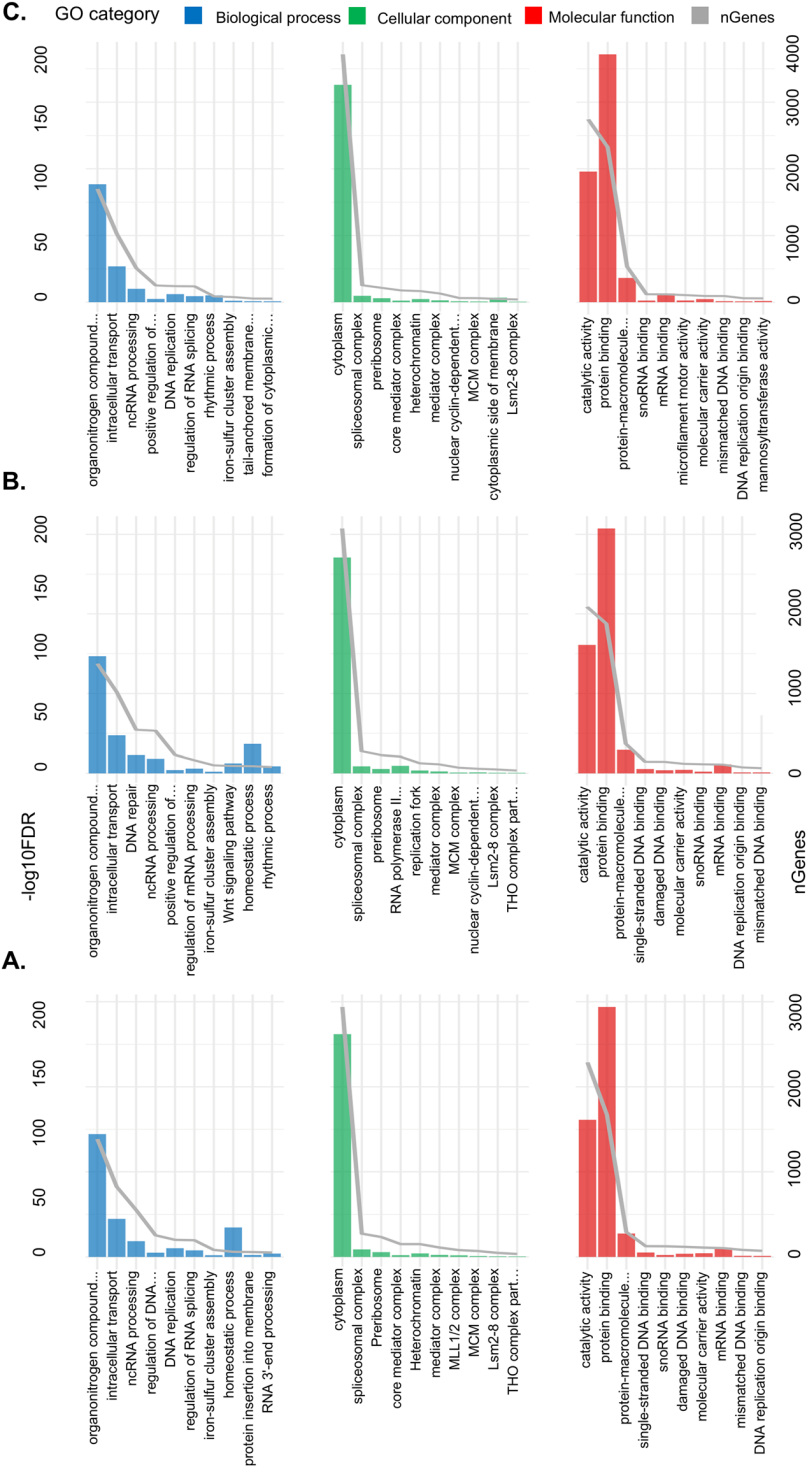
GO term enrichment profiles used to validate annotation completeness. Top ten pathways from each main GO category are shown for the German (DE), Swedish (SWE), and combined (DE-SWE) assemblies. Bars indicate gene counts and enrichment significance (–log10 FDR).

To evaluate the diversity of functional categories captured by the annotation, we performed Gene Ontology (GO) enrichment analyses. The GO enrichment analysis and classification of transcripts across the three assemblies (DE, SWE, and DE-SWE) reveals key differences and similarities in functional categories (Fig. 7). Molecular Function category was dominated by “catalytic activity” and “protein binding,” with DE showing 1,612 and 2,939 genes, SWE 1,610 and 3,073 genes, and DE-SWE 1,958 and 3,716 genes, respectively. Enriched functions such as “protein-macromolecule adaptor activity” and “single-stranded DNA binding” underline molecular interactions central to cellular processes. For the Cellular Component, “cytoplasm” had the highest enrichment, showing substantial gene counts in each assembly (i.e., 2,619 in DE, 2,709 in SWE, and 3,259 in DE-SWE). “Spliceosomal complex” was also prominent, involving 88 genes in both DE and SWE, and 95 in DE-SWE. Other enriched components included “preribosome,” “core mediator complex,” and “mediator complex,” indicating shared cellular structures across datasets. Finally, for Biological Function, “organonitrogen compound metabolic process” was the most enriched across all assemblies, with the DE assembly containing 1,444 genes, SWE 1,468 genes, and DE-SWE 1,768 genes. These categories confirm that key biological processes are well captured, serving as evidence of annotation completeness and diversity.

### KEGG pathway analysis for functional coverage validation

**Fig. 8:**
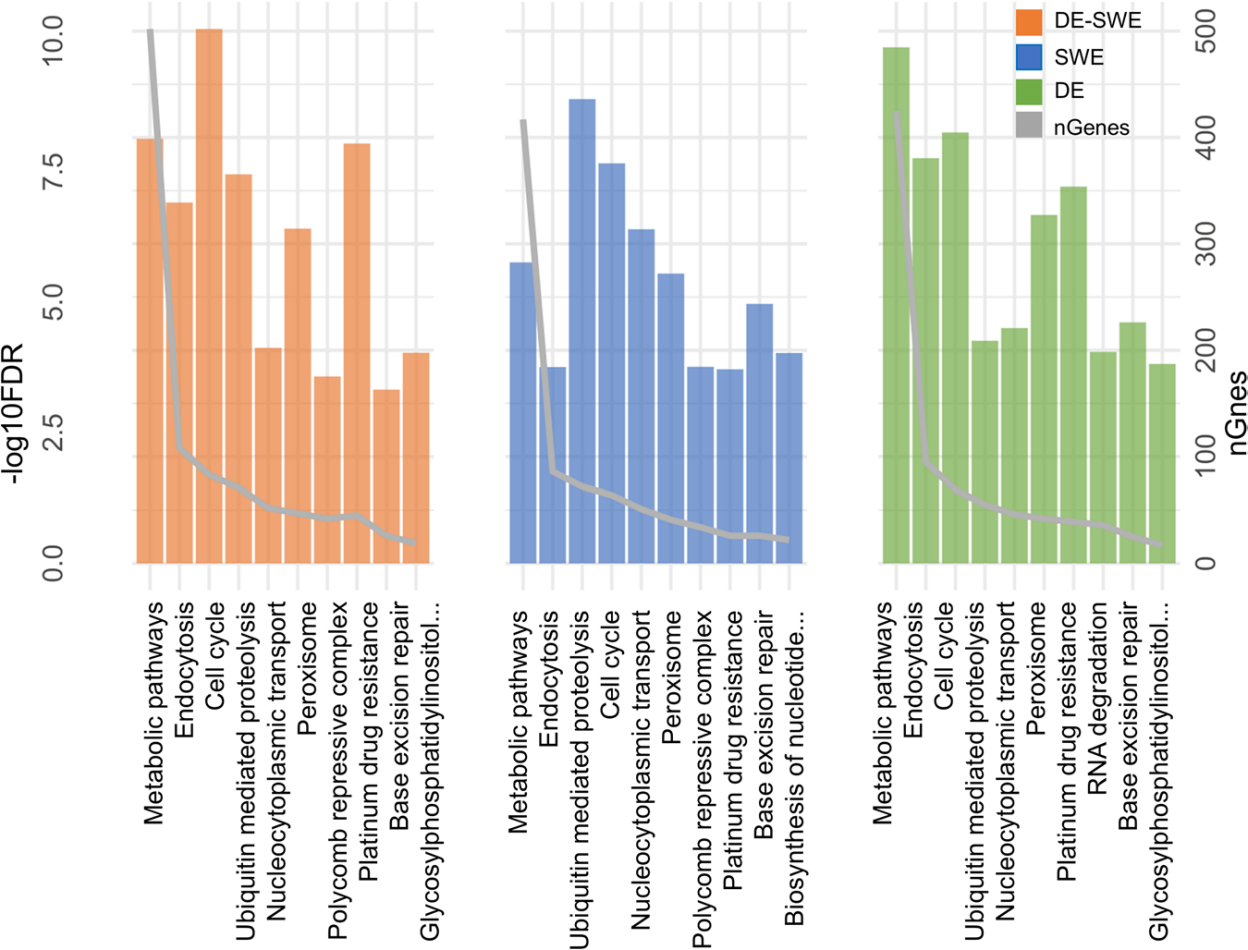
Top KEGG pathways used for assessing functional diversity in the annotation. Enriched pathways are shown with gene counts and enrichment significance (–log10 FDR) for the German (DE), Swedish (SWE), and combined (DE-SWE) assemblies.

The top ten KEGG pathways from the enrichment analysis (Fig. 8), reveal shared enrichment trends across the DE, SWE, and DE-SWE assemblies, with metabolic pathways and the cell cycle pathway highly enriched in all three. Metabolic pathways involved 425 genes in DE, 417 in SWE, and 502 in DE-SWE, while the cell cycle pathway included 69 genes in DE, 72 in SWE, and 83 in DE-SWE. Distinct enrichments appeared in each assembly: endocytosis was prominent in DE-SWE (108 genes) compared to DE (95) and SWE (86), and pathways like platinum drug resistance (45 genes) and the polycomb repressive complex (42 genes) were more enriched in DE-SWE. Ubiquitin-mediated proteolysis showed variability, with 71 genes in DE-SWE, 55 in DE, and 64 in SWE. These results reflect the functional diversity of the assemblies and serve to validate the completeness and utility of the transcriptomes for future functional genomics applications.

## Supplementary information

Table S1.

## Data Availability

No specialized code was used in this research. The software programs employed for *de novo* transcriptome assembly, pre- and post-assembly steps, and transcriptome annotation are listed with their respective versions in the Methods section. Where specific parameters are not provided, the programs were run using their default settings.
