# Supplementary material for: De novo transcriptome assembly and annotation of the common freshwater amphipod (Gammarus pulex) a valuable resource for ecotoxicogenomics
Source: Sci Data. 2025 Aug 28;12:1502. doi: 10.1038/s41597-025-05872-2 (PMC12394684; doi:10.1038/s41597-025-05872-2)
Supplement: Supplementary file 1 — Table S1. [file 41597_2025_5872_MOESM1_ESM.pdf]

Supplementary Information Contents

Page 2: Table S1. Sample metadata and accession information for RNA-seq libraries from Gammarus pulex.

Table S1. Sample metadata and accession information for RNA-seq libraries from *Gammarus pulex.*

This table lists internal sample names, collection dates, geographic coordinates, and corresponding NCBI accessions (SRA run, BioSample, and Experiment) for specimens collected in Sweden and Germany.

sample_name	country	collection_date	lat_wgs84	lon_wgs84	sra_run_accession	biosample_accession	experiment_accession
DE_01	Germany	2021-10-29	51.849829	10.797051	SRR32767953	SAMN47145853	SRX28052682
DE_02	Germany	2021-10-29	51.849829	10.797051	SRR32767952	SAMN47145854	SRX28052683
DE_03	Germany	2021-10-29	51.849829	10.797051	SRR32767951	SAMN47145855	SRX28052684
DE_04	Germany	2021-10-29	51.849829	10.797051	SRR32767950	SAMN47145856	SRX28052685
DE_05	Germany	2021-10-29	51.849829	10.797051	SRR32767949	SAMN47145857	SRX28052686
DE_06	Germany	2021-10-29	51.867888	10.873541	SRR32767947	SAMN47145858	SRX28052688
DE_07	Germany	2021-10-29	51.867888	10.873541	SRR32767946	SAMN47145859	SRX28052689
DE_08	Germany	2021-10-29	51.867888	10.873541	SRR32767945	SAMN47145860	SRX28052690
DE_09	Germany	2021-10-29	51.867888	10.873541	SRR32767944	SAMN47145861	SRX28052691
DE_10	Germany	2021-10-29	51.867888	10.873541	SRR32767943	SAMN47145862	SRX28052692
DE_11	Germany	2021-10-30	51.885029	10.963073	SRR32767942	SAMN47145863	SRX28052693
DE_12	Germany	2021-10-30	51.885029	10.963073	SRR32767941	SAMN47145864	SRX28052694
DE_13	Germany	2021-10-30	51.885029	10.963073	SRR32767940	SAMN47145865	SRX28052695
DE_14	Germany	2021-10-30	51.885029	10.963073	SRR32767939	SAMN47145866	SRX28052696
DE_15	Germany	2021-10-30	51.885029	10.963073	SRR32767938	SAMN47145867	SRX28052697
DE_16	Germany	2021-10-29	51.904336	11.061795	SRR32767936	SAMN47145868	SRX28052699
DE_17	Germany	2021-10-29	51.904336	11.061795	SRR32767935	SAMN47145869	SRX28052700
DE_18	Germany	2021-10-29	51.904336	11.061795	SRR32767934	SAMN47145870	SRX28052701
DE_19	Germany	2021-10-29	51.904336	11.061795	SRR32767933	SAMN47145871	SRX28052702
DE_20	Germany	2021-10-29	51.904336	11.061795	SRR32767932	SAMN47145872	SRX28052703
DE_21	Germany	2021-10-29	51.905442	11.067436	SRR32767931	SAMN47145873	SRX28052704
DE_22	Germany	2021-10-29	51.905442	11.067436	SRR32767930	SAMN47145874	SRX28052705
DE_23	Germany	2021-10-29	51.905442	11.067436	SRR32767929	SAMN47145875	SRX28052706
DE_24	Germany	2021-10-29	51.905442	11.067436	SRR32767928	SAMN47145876	SRX28052707
DE_25	Germany	2021-10-29	51.905442	11.067436	SRR32767927	SAMN47145877	SRX28052708
DE_26	Germany	2021-10-30	51.941626	11.158676	SRR32767925	SAMN47145878	SRX28052710
DE_27	Germany	2021-10-30	51.941626	11.158676	SRR32767924	SAMN47145879	SRX28052711
DE_28	Germany	2021-10-30	51.941626	11.158676	SRR32767923	SAMN47145880	SRX28052712
DE_29	Germany	2021-10-30	51.941626	11.158676	SRR32767922	SAMN47145881	SRX28052713
DE_30	Germany	2021-10-30	51.941626	11.158676	SRR32767921	SAMN47145882	SRX28052714

SW_01	Sweden	2021-07-14	55.575026	13.700139	SRR32767982	SAMN47145818	SRX28052653
SW_02	Sweden	2021-07-14	55.575026	13.700139	SRR32767981	SAMN47145819	SRX28052654
SW_03	Sweden	2021-07-14	55.575026	13.700139	SRR32767970	SAMN47145820	SRX28052665
SW_04	Sweden	2021-07-14	55.575026	13.700139	SRR32767959	SAMN47145821	SRX28052676
SW_05	Sweden	2021-07-14	55.575026	13.700139	SRR32767948	SAMN47145822	SRX28052687
SW_06	Sweden	2021-07-13	55.432667	13.454389	SRR32767937	SAMN47145823	SRX28052698
SW_07	Sweden	2021-07-13	55.432667	13.454389	SRR32767926	SAMN47145824	SRX28052709
SW_08	Sweden	2021-07-13	55.432667	13.454389	SRR32767920	SAMN47145825	SRX28052715
SW_09	Sweden	2021-07-13	55.432667	13.454389	SRR32767919	SAMN47145826	SRX28052716
SW_10	Sweden	2021-07-13	55.432667	13.454389	SRR32767918	SAMN47145827	SRX28052717
SW_11	Sweden	2021-07-15	55.687944	13.077833	SRR32767980	SAMN47145828	SRX28052655
SW_12	Sweden	2021-07-15	55.687944	13.077833	SRR32767979	SAMN47145829	SRX28052656
SW_13	Sweden	2021-07-15	55.687944	13.077833	SRR32767978	SAMN47145830	SRX28052657
SW_14	Sweden	2021-07-15	55.687944	13.077833	SRR32767977	SAMN47145831	SRX28052658
SW_15	Sweden	2021-07-15	55.687944	13.077833	SRR32767976	SAMN47145832	SRX28052659
SW_16	Sweden	2021-07-14	55.855224	12.927046	SRR32767975	SAMN47145833	SRX28052660
SW_17	Sweden	2021-07-14	55.855224	12.927046	SRR32767974	SAMN47145834	SRX28052661
SW_18	Sweden	2021-07-14	55.855224	12.927046	SRR32767973	SAMN47145835	SRX28052662
SW_19	Sweden	2021-07-14	55.855224	12.927046	SRR32767972	SAMN47145836	SRX28052663
SW_20	Sweden	2021-07-14	55.855224	12.927046	SRR32767971	SAMN47145837	SRX28052664
SW_21	Sweden	2021-07-14	55.591297	13.422503	SRR32767969	SAMN47145838	SRX28052666
SW_22	Sweden	2021-07-14	55.591297	13.422503	SRR32767968	SAMN47145839	SRX28052667
SW_23	Sweden	2021-07-14	55.591297	13.422503	SRR32767967	SAMN47145840	SRX28052668
SW_24	Sweden	2021-07-14	55.591297	13.422503	SRR32767966	SAMN47145841	SRX28052669
SW_25	Sweden	2021-07-14	55.591297	13.422503	SRR32767965	SAMN47145842	SRX28052670
SW_26	Sweden	2021-07-14	55.683906	13.179930	SRR32767964	SAMN47145843	SRX28052671
SW_27	Sweden	2021-07-14	55.683906	13.179930	SRR32767963	SAMN47145844	SRX28052672
SW_28	Sweden	2021-07-14	55.683906	13.179930	SRR32767962	SAMN47145845	SRX28052673
SW_29	Sweden	2021-07-14	55.683906	13.179930	SRR32767961	SAMN47145846	SRX28052674
SW_30	Sweden	2021-07-14	55.683906	13.179930	SRR32767960	SAMN47145847	SRX28052675
SW_31	Sweden	2021-07-13	55.448964	13.592180	SRR32767958	SAMN47145848	SRX28052677
SW_32	Sweden	2021-07-13	55.448964	13.592180	SRR32767957	SAMN47145849	SRX28052678
SW_33	Sweden	2021-07-13	55.448964	13.592180	SRR32767956	SAMN47145850	SRX28052679
SW_34	Sweden	2021-07-13	55.448964	13.592180	SRR32767955	SAMN47145851	SRX28052680
SW_35	Sweden	2021-07-13	55.448964	13.592180	SRR32767954	SAMN47145852	SRX28052681
